# Peripheral nerve stimulator for terminal sciatic nerve neuromas in an amputee

**DOI:** 10.3171/2020.7.FOCVID2029

**Published:** 2020-10-01

**Authors:** Lekhaj C. Daggubati, Justin R. Davanzo, Elias B. Rizk

**Affiliations:** Department of Neurosurgery, The Pennsylvania State University, College of Medicine, Hershey, Pennsylvania

**Keywords:** peripheral nerve stimulator, neuroma, neuromodulation, postamputation pain

## Abstract

Neuromas are a difficult-to-treat peripheral nerve pathology that can cause crippling pain. Optimal treatment is widely debated as pharmacological intervention frequently is not sufficient and surgical interventions are plagued with recurrence. The majority of amputees report severe and chronic stump pain. Avoiding complex surgery at the stump site would prevent infection or wound dehiscence. Recent advances in neuromodulation with external pulse emitters allow for pain relief with localized nerve stimulation. The authors describe the novel placement of a sciatic nerve stimulator in a 77-year-old man for painful stump neuromas of the common peroneal and tibial nerves.

The video can be found here: https://youtu.be/96kKs3qjtqc

## Transcript

**0:20 Case Presentation.** We have the pleasure of presenting our case using a peripheral nerve stimulator for terminal sciatic nerve neuromas in a 77-year-old amputee.

**0:30** This is a 77-year-old male with a history of right lower-extremity amputation in 1993 that has been experiencing 4 years of localized right lower-extremity pain since a right femur fracture requiring an orthopedic surgery. The pain is localized to the terminal medial portion of his stump. He has attempted and failed conservative treatment with gabapentin, lidocaine patches, and local injections. An external trial was performed in clinic and it gave him complete resolution. So, he presented for permanent implantation.

**1:08** To briefly summarize, neuromas are difficult-to-treat painful entities caused by peripheral nerve trauma.^1^ There are multiple pharmacological and surgical interventions used. For nerve-end neuromas, some of the popular treatments include burying the nerve in either bone or muscle or capping the nerve end.^2,3^ However, the long-term data is limited. And recurrence and reoperation has been reported to be as high as 65%. In addition, about 20% to 30% of neuromas do not respond to treatment.^4^ In the amputee population, the prevalence of stump pain is above 75%.^5^

**1:48** Peripheral nerve stimulators prove to be a minimally invasive option for these patients. Neuromodulation gives the advantage of physiologically changing sensory perception without anatomical alterations.^6^ Recent technology in radiofrequency has allowed for external pulse emitters instead of the standard implantable pulse generators. This allows for transdermal deliverance of the stimulation to the implanted electrode. It also allows for a lower-profile, targeted stimulator placement without being tethered to a generator.^7^ It avoids potentially unnecessary back and bilateral lower-extremity stimulation associated with spinal cord stimulators. And more importantly, this does not preclude further surgical intervention if the peripheral nerve stimulator proves to be ineffective.

**2:38 Preoperative Imaging.** On imaging, we can see on this T1 coronal fat-saturated MRI of his right leg that he has two terminal neuromas of his sciatic nerve. The cranial one is a common peroneal nerve neuroma, while the caudal one is a tibial nerve neuroma.

**2:55 Patient Positioning.** To allow for nerve localization, the patient is consented for an awake, prone procedure. He is allowed to lay on his abdomen under mild sedation. We had drawn out the anticipated route of the sciatic nerve as well as an entry site toward the sciatic nerve from a lateral approach. There is also a tunneling site superior to it that was marked.

**3:17** Under ultrasound, we localize the sciatic nerve, which in this image is shown as a hyperechoic speculated structure.

**3:26** This image shows all of the instruments that are used during the case, and we will run through them as they show up in the video.

**3:34 Sciatic Nerve Localization.** After the patient was prepped and draped, the ultrasound is used to localize the sciatic nerve again and an angiocath needle is placed at the lateral entry site. The needle is removed and, with the angiocath sheath in place, a localizer electrode is placed under ultrasound until it is abutting the sciatic nerve. At this point, a wire with an electrical current is connected to the end of the localizer to confirm the patient is having sensation at the site of his pain.

**4:06** Once confirmed, the angiocath sheath is removed and, carefully, an 18-gauge trocar is placed over the localizer wire. Previous experience has shown us to mark the trocar with the depth shown on the localizer electrode. In this case, it is approximately 8½ cm from the entry to the sciatic nerve. As seen, the patient can also give us feedback when we are at the sciatic nerve. During the trocar placement, the localizer electrode can sometimes be pushed out. We push it back in until mild resistance is felt. And this is once again connected to the wire to confirm appropriate placement.

**4:49 Final Lead Placement.** It is vital to check after every step that we are still next to the sciatic nerve. The localizer electrode and inner cannula of the trocar are removed with the trocar outer sheath in place. After confirming that the sheath is still at the predetermined mark, the final lead and a casing are placed into the trocar sheath.

**5:14** The trocar is backed out slightly to ensure the electrode is in touch with the sciatic nerve. And at this point, we reconfirmed appropriate stimulation by attaching it to the wire. Keeping the wire in place, we use the Seldinger method to remove the sheath from around the final electrode and its casing. Care should be taken to make sure the electrode remains in place through this process. As always, we check to ensure the patient has appropriate stimulation at the site of his pain once the sheath is removed.

**5:51 Casing Removal and Electrode Tunneling.** Using forceps, the inner electrode within the casing is pushed forward slightly to ensure the anchors engage the soft tissue. Afterwards, you slowly pull back on the casing, making sure the electrode does not move and the anchors are appropriately engaged. Once out, this will expose the distal portion of the final electrode, which is always connected to the outside current to ensure appropriate placement. Then, we use the tunneler to tunnel down to the initial entry site, making sure not to damage the electrode. The distal portion is then passed through the cannula of the tunneler and the tunneler is pulled out from its tunneling site. This allows for the entire electrode to be below the dermis.

**6:49** Postoperative Care. Postoperatively, the patient did well and discharged the same day. The peripheral nerve stimulator is programmed prior to discharge. He is able to attach the cutaneous external pulse emitter over the distal portion of his electrode. One month out from surgery, the patient reports significant pain relief. He has resumed playing golf, and the stimulator use has improved his sleep.

**7:20 References**

## Author Contributions

Primary surgeon: Rizk. Assistant surgeon: Daggubati, Davanzo. Editing and drafting the video and abstract: Daggubati, Davanzo. Critically revising the work: Daggubati, Davanzo. Reviewed submitted version of the work: Daggubati, Davanzo.
